# How to Hold Our Husbands

**Published:** 1913-04

**Authors:** 


### How To Hold Our Husbands.

An article under this title, written by a wife and mother, has appeared twice in the "Red Back," and has been copied in several other periodicals. It has created a great deal of comment from earnest, thinking people. Most of the critics have lauded the article, while some have severely criticised it. Those who have spoken adversely of it, in the main, have misinterpreted the author's purpose and meaning, charging her with compromising the self-respect of woman and as regarding her in the light of a slave to man's lust. We take it that nothing was further from the author's thoughts, as her plea was for the sanctity of the home, and an endeaver to show some women how the power they possess in their sexual natures, might be properly used to cement more deeply the matrimonial bond. There was no intention of holding woman as debased, or in the relation of prostitution, as she was pleading with normal men and women, where self-respect was supposed to exist as a matter of course. Therefore, any allusion to woman's abasement has no place in the argument. With the home held inviolate, as the unit of society, the further purpose of the author was to show how this institution might be preserved from dissolution, by the wife "coming into her own," not in a subservient capacity, but in the exercise of her full right, founding her premises, as the author does, on the biological bases of the sex relation, as the real foundation of the home. It was this attitude of the question that commended it to the critics who praised the article. They seem to think it rather remarkable that a woman should have such a deep grasp of so fundamental a question.

The contention of those who hold to the modern economic independence of women, which would favor a greater freedom of divorce, represents the academic argument of the question, based on love as the sole "tie that binds," and as being above law or church. This in all probability will be the rule in the future, judging by the signs of the times. But it cannot be gainsaid that our great modern civilization has been developed under the old regime, and we are not without some misgivings as to what the new order of things will develop.

Always, in discussing this subject, it should be remembered that it is not the dominance of one party over the other, or the question of special privilege, but the mutual sacrifice on the part of both for the happiness of each other. We must discriminate between lust and love as applied to the relations of man and woman. If this attitude between the sexes were regarded, and a greater knowledge of the higher purpose of sex instinct were imparted and fortified by the eugenic principle of proper mating, the next generation would have no grounds for divorce.

